# Effect of surgical pleth index-guided remifentanil administration on perioperative outcomes in elderly patients: a prospective randomized controlled trial

**DOI:** 10.1186/s12871-023-02011-5

**Published:** 2023-02-17

**Authors:** Young Ju Won, Seok Kyeong Oh, Byung Gun Lim, Young Sung Kim, Do Yeop Lee, Jae Hak Lee

**Affiliations:** Department of Anesthesiology and Pain Medicine, Korea University Guro Hospital, Gurodong-Ro 148, Guro-Gu, Seoul, 08308 Republic of Korea

**Keywords:** Surgical pleth index, Opioid analgesics, Nociception, Aged, Delirium, NK cell activity

## Abstract

**Background:**

During general anesthesia, the surgical pleth index (SPI) monitors nociception. The evidence of SPI in the elderly remains scarce. We aimed to investigate whether there is a difference in perioperative outcomes following intraoperative opioid administration according to the surgical pleth index (SPI) value versus hemodynamic parameters (heart rate or blood pressure) in elderly patients.

**Methods:**

Patients aged 65–90 years who underwent laparoscopic colorectal cancer surgery under sevoflurane/remifentanil anesthesia were randomized to receive remifentanil guided by SPI (SPI group) or conventional clinical judgment based on hemodynamic parameters (conventional group). The primary endpoint was intraoperative remifentanil consumption. Secondary endpoints were intraoperative hemodynamic instability, pain score, fentanyl consumption and delirium in the post-anesthesia care unit (PACU), and perioperative changes in interleukin-6 and natural killer (NK) cell activity.

**Results:**

Seventy-five patients (38, SPI; 37, conventional) were included in the study. The SPI group consumed significantly more remifentanil intraoperatively than the conventional group (mean ± SD, 0.13 ± 0.05 vs. 0.06 ± 0.04 μg/kg/min, *P* < 0.001). Intraoperative hypertension and tachycardia were more common in the conventional group than in the SPI group. Pain score in the PACU (*P* = 0.013) and the incidence of delirium in the PACU were significantly lower in the SPI group than the conventional group (5.2% vs. 24.3%, *P* = 0.02). There was no significant difference in NK cell activity and interleukin-6 level.

**Conclusions:**

In the elderly patients, SPI-guided analgesia provided appropriate analgesia with sufficient intraoperative remifentanil consumption, lower incidence of hypertension/ tachycardia events, and a lower incidence of delirium in the PACU than the conventional analgesia. However, SPI-guided analgesia may not prevent perioperative immune system deterioration.

**Trial registration:**

The randomized controlled trial was retrospectively registered in the UMIN Clinical Trials Registry (trial number: UMIN000048351; date of registration: 12/07/2022).

## Background

Surgical procedures cause profound physiological changes, including hemodynamic, endocrine, and immune functions [1]. However, because the patient cannot complain of pain during general anesthesia and the autonomic nervous system homeostasis also decreases, proper analgesia must be provided by monitoring intraoperative noxious stimulation.

Moreover, in elderly patients, homeostatic balance is more easily altered by exogenous stimuli [2]. Altered homeostatic balance due to trauma may lead to impaired immune function, which can be more critical in elderly patients who undergo cancer surgery [3, 4]. Despite rising patient’s expectations for good surgical outcomes due to the development of surgical, anesthetic, and medical techniques, elderly patients continue to suffer from postoperative adverse outcomes as they age.

Monitoring devices for estimating the effect of nociception during general anesthesia have recently become commercially available [5, 6]. The surgical pleth index (SPI) is a monitoring tool for detecting the balance between nociceptor activation and analgesia during general anesthesia derived from photoplethysmographic signals of the finger arteriole [7]. The SPI values are calculated using the following equation: $\mathrm{SPI}\:=\:100\;-\;(0.33\:\times\:\mathrm{HBI}\:+\:0.67\:\times\:\mathrm{PPGA})$ [HBI: heart beat interval and PPGA: photoplethysmographic waveform amplitude] [8]. The SPI enabled proper analgesia with less intraoperative opioid consumption [9]. However, these findings were based on relatively healthy non-older adult patients: no studies have been conducted to investigate the usefulness or range of SPIs in providing adequate analgesia during surgery in elderly patients. The usefulness of SPI in this population might be questionable due to the differences in vascular properties, such as arterial stiffness and elasticity between elderly and non-elderly patients.

Therefore, this randomized controlled trial aimed to investigate whether there is a change in the perioperative outcomes caused by a difference in opioid administrations during surgery according to the SPI value versus hemodynamic parameters such as heart rate (HR) or blood pressure (BP) in elderly patients undergoing laparoscopic colorectal surgery. In addition, changes in inflammatory and immunologic markers were investigated for homeostatic imbalance analysis. We hypothesized that SPI-guided intraoperative analgesia would reduce intraoperative remifentanil consumption, allowing for earlier awakening and better immune function with a similar pain level after surgery.

## Methods

### Ethical approval

**Fig. 1:**
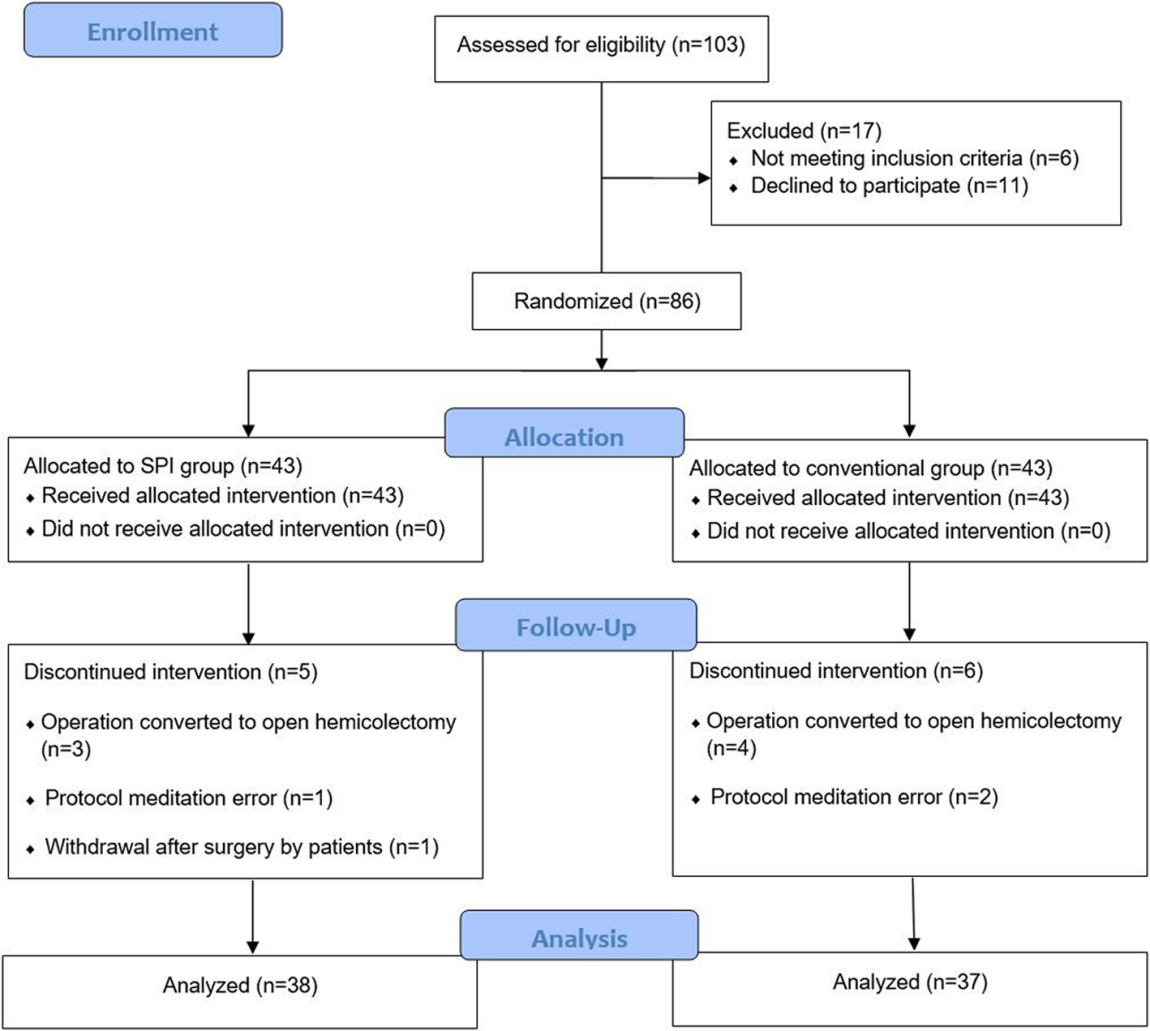
Flow chart showing patient recruitment, random assignment, and withdrawal

The Korea University Guro Hospital Institutional Review Board (2019GR0178) approved this study, and it was retrospectively registered in the UMIN Clinical Trials Registry (trial number: UMIN000048351; receipt number: R000055068; date of registration: 12/07/2022). The study was conducted between May 2019 and March 2021 in a single university teaching hospital (Korea University Guro Hospital, Seoul, South Korea) and followed the Consolidated Standards of Reporting Trials (CONSORT) 2010 guidelines (Fig. 1). All patients and/or their legal guardian(s) provided written informed consent prior to data collection or study intervention.

### Participants and randomization

All patients were recruited from the Department of Colorectal Surgery, Korea University Guro Hospital by the research staff.

The inclusion criteria were as follows: patients scheduled to undergo elective laparoscopic colorectal cancer surgery, between the ages of 65 and 90 years, with an American Society of Anesthesiologists (ASA) physical status I–III. The exclusion criteria were as follows: history of neuromuscular, neurological, or psychological diseases (i.e., dementia, Parkinson’s disease, seizure disorders, head injury); abuse of alcohol or illicit drugs or chronic use of psychoactive medication; significant cardiac arrhythmia (i.e., atrial fibrillation, or atrioventricular block exceeding 1^st^ degree);hemodynamically unstable condition before surgery; those with an allergic history to medication used in the study; those with difficulty communicating owing to hearing or pronunciation problems; those who showed severe anxiety before entering the operation room; and those who could not cooperate.

Patients were randomly assigned to two groups (1:1 ratio) without being aware of the assigned group to receive remifentanil infusion guided by SPI (SPI group) or by clinical judgment based on hemodynamic parameters (conventional group). Patients were assigned at random by a single investigator. A web-based (www.randomization.com) computer-generated list was used for randomization. The random numbers were kept in opaque, sealed envelopes until they were opened in the operating room by an independent anesthesiologist who was not involved in the study.

### Anesthetic management

No premedication was administered before the surgery. Because of the patient’s circadian rhythm, all operations began before 11:30 a.m. All patients in the operating room were fitted with intraoperative monitoring devices (e.g., devices for non-invasive arterial BP measurement, pulse oximeter [CARESCAPE monitor B650, GE Healthcare Finland Oy, Finland], electrocardiogram, and entropy monitor [GE Healthcare, Helsinki, Finland]). Forced-air warming blankets were given to all patients to minimize perioperative hypothermia. Before anesthesia induction, a 20 G cannula was inserted into the radial artery after intradermal injection of 2% lidocaine to the contralateral arm, where an intravenous line was placed, and the first blood sampling was performed using the arterial line. In all patients a pulse oximeter sensor for SPI measurement was attached to the index finger of the hand with the intravenous catheter. Prior to anesthesia induction, the baseline values for mean SPI, HR, and mean arterial pressure (MAP) were recorded. All patients were administered balanced anesthesia with sevoflurane and remifentanil. For the induction of general anesthesia, the patients were administered propofol 2 mg/kg and target-controlled infusion (TCI) of remifentanil with target effect-site concentration (Ce_remi_) of 4 ng/mL via a TCI pump (INJECTOMAT® MC AGILIA; Fresenius Kabi, Bad Homburg, Germany), and the pharmacokinetic model of Minto and colleagues was used for remifentanil TCI [10]. After the loss of consciousness, each patient was injected with rocuronium 0.6 mg/kg, and their trachea was intubated. Ventilation was adjusted with 50% oxygen in the air to maintain a partial pressure of end-tidal carbon dioxide between 30 and 35 mmHg. Sevoflurane was administered to all patients at starting concentration of 2 vol% and gradually adapted to maintain a state entropy level of 40–60.

Using the previously described methodology, the SPI was automatically calculated on the monitor used in the study [8]. SPI values range from 0 to 100, with higher scores indicating higher stress levels. A value of 50 indicates the average stress level during anesthesia, and an SPI range between 20 and 50 is adopted to guide adequate analgesia during anesthesia.

The signs of inadequate anesthesia were defined as follows: somatic movement, such as grimacing, coughing, and effort of breathing, and hemodynamic events, such as tachycardia and hypertension.

A rescue medication (additional propofol 20 mg) was administered in both groups when signs of inadequate anesthesia are identified such as somatic movements, such as grimacing, coughing, and effort of breathing during surgery despite state entropy, MAP, HR, or SPI values within the predefined normal range. Events of Hemodynamic instability were identically defined in both groups. Tachycardia was defined as an HR of > 90 beats/min, and hypertension was defined as MAP > 100 mmHg if the baseline MAP was < 83 mmHg or an increase of over 20% of the baseline MAP if it was > 83 mmHg. Bradycardia was defined as HR < 45 beats/min. Hypotension was defined as MAP < 60 mmHg, as previous study’s definition [11].

In the SPI group, the Ce_remi_ was regulated to keep the SPI values between 20 and 50 by increasing (if SPI > 50) or decreasing (if SPI < 20) remifentanil by 0.5 ng/mL, from 0 to 5 ng/mL of Ce_remi,_ and by 1 ng/ml from 5 to 10 ng/mL of Ce_remi_ every 1 min in a stepwise manner (Ce_remi_ range: 0 and 10 ng/mL). If hypotension persisted with SPI of 20–50, 200 mL crystalloid was administered, and ephedrine 4 mg was administered at a 5-min interval, maximally twice in an instance. If hypotension persisted despite two continuous ephedrine administrations, phenylephrine infusion was commenced. If hypertension occurred despite an SPI value below 50, nicardipine 0.5 mg was administered at a 5-min interval.

In the conventional group, the signs of inadequate anesthesia were treated by gradually increasing remifentanil (0.5 ng/mL) from 0 to 5 ng/mL of Ce_remi_, and by 1 ng/ml from 5 to 10 ng/mL of Ce_remi_ every 1 min in a stepwise manner (Ce_remi_ range: 0 and 10 ng/mL). Hypotension was treated initially by decreasing remifentanil by 0.5 ng/mL Ce_remi_ every 1 min in a stepwise manner; if two stepwise remifentanil reductions were ineffective for treating hypotension, 200 mL crystalloid was administered, and ephedrine 4 mg was administered, and repeated twice within 10 min, if we concluded that hypotension persisted. Thereafter, if hypotension persisted, phenylephrine infusion was initiated. If hypertension or tachycardia persisted despite two sequential remifentanil increases (total, 1–2 ng/mL) within 3 min, nicardipine (0.5 mg) was administered at a 5-min interval.

SPI, MAP, and HR were recorded just before induction, 1 min, 5 min after intubation, and 5-min intervals from trocar insertion until the end of skin suture. Operation time was defined as the time after trocar insertion up to skin suture. For proper neuromuscular blockade, the neuromuscular function was monitored with acceleromyography using the TOF-Watch SX® (Organon Ireland Ltd, Schering-Plough Corporation, Dublin, Ireland). Rocuronium (5 mg) was additionally applied at the reappearance of T1 (the first response of train-of-four [TOF] stimulation) just until the specimens were removed.

Skin suturing was performed after administration of ramosetron 3 mg i.v. for preventing nausea and vomiting. Then, paracetamol 1 g was administered every 8 h intravenously to control postoperative pain in all patients up to 48 h. At the end of the surgery, the administration of sevoflurane and remifentanil was stopped, the fresh gas flow was increased to 8 L/min of oxygen, and sugammadex 2 mg/kg was administered to reverse neuromuscular blockade after confirming a TOF count of 2.

### Post-operative management and assessment

Extubation was performed after the patient regained consciousness and spontaneous breathing. The patient was transferred to the post-anesthesia care unit (PACU). Awakening time was defined as the time from the discontinuation of anesthetics to eye opening and assessed by a blinded independent anesthesiologist.

The blinded anesthesiologist in the PACU measured the modified Aldrete score every 10 min for 60 min, the numerical rating scale (NRS; 0–10) for pain every 10 min for 60 min, the cumulative consumption of rescue fentanyl, and the occurrence of adverse events, including nausea or vomiting. Fentanyl 25 μg was administered in the PACU for postoperative pain control, for an NRS score ≥ 6, and the treatment was repeated at 10-min intervals. Metoclopramide hydrochloride 10 mg was administered for nausea and vomiting. Delirium was defined as positive in the PACU using the CAM-ICU (confusion assessment method for the intensive care unit) tool, and it was assessed before transferring from PACU to the ward.

In the ward, the blinded anesthesiologist assessed the following outcomes, NRS for pain on postoperative day [POD] 1, cumulative pethidine consumption on POD 1, and postoperative length of hospital stay. Pethidine 25 mg was injected for an NRS score ≥ 5 in the ward.

### NK cell activity and IL-6 measurement

Blood samples for measuring inflammatory and immunologic markers (interleukin [IL]-6, natural killer [NK] cell activity) were collected at baseline (preoperative) and 24 h after surgery.

A blood test kit (NK Vue® Kit, ATGen, Sungnam, Republic of Korea) was used to measure NK cell activity. Briefly, a 1 ml sample of whole blood was transferred into a specific tube for the NK cell activity test, containing a patented stimulatory cytokine (Promoca™, ATGen, Sungnam, Republic of Korea). The collection tube was repeatedly and gently mixed; within 30 min of collection, the tube was incubated for approximately 22 h in a 37 °C chamber, according to the manufacturer’s instructions. The stimulatory cytokine produces interferon-γ into the plasma during the incubation period; this secretion predominantly occurs via NK cells rather than innate adaptive immune cells [12]. After incubation, the supernatant was collected and centrifuged at 3000 × g for 3 min. The supernatant was immediately loaded onto an enzyme-linked immunosorbent assay (ELISA) plate. Using a designed ELISA, interferon-γ levels were quantitated and expressed in pg/mL. Proinflammatory cytokine IL-6 was measured in serum using a commercial ELISA kit (D6050, R&D Systems, MN, USA). The absorbance was assessed using a SpectraMax® 190 microplate reader (Molecular Devices, China).

### Statistical analysis

The Primary endpoint was intraoperative remifentanil consumption. Secondary endpoints include intraoperative hemodynamic instability events, awakening time, pain score, fentanyl consumption and incidence of delirium in the PACU, and perioperative changes in inflammatory and immunologic markers, including IL-6 and NK cell activity.

As intraoperative remifentanil consumption in both groups was the primary endpoint in this study, the sample size was determined based on the results of the opioid consumption (mean ± SD = 3.5 ± 2.4 vs. 5.1 ± 2.4 mg in the SPI vs. the conventional groups) in a previous report by Won et al. [11], using G*Power software, version 3.1 (Franz Faul, Universität Kiel, Kiel, Germany). Therefore, the effect size of the two groups was 0.66. On the assumption that the allocation ratio of the two groups was 1, a sample size of 37 patients was calculated for each group by Student and two-sided t-tests with a significance level of 0.05 and a power of 0.8. We estimated a 15% dropout rate, resulting in a final enrolment of 43 patients in each group.

The SPSS software version 18 (SPSS Inc., IBM, Chicago, IL, USA) was used for statistical analysis. Continuous data included age, weight, height, anesthesia time, awakening time_,_ intraoperative remifentanil consumption, rescue fentanyl consumption in the PACU, NRS for pain in the PACU, NRS for pain on POD 1. A two-tailed student’s t-test (normally distributed data) or Mann–Whitney U test (abnormally distributed data) was used to analyze cumulative pethidine consumption on POD 1 and postoperative length of hospital stay between the groups. A Chi-squared test or Fisher’s exact test was used to compare the categorical data (preoperative medication use and medical histories such as diabetes mellitus [DM], hypertension, calcium channel blockers, beta-blockers, angiotensin II receptor blockers, angiotensin-converting enzyme inhibitors, preoperative chemotherapy, sex, intraoperative phenylephrine infusion use, intraoperative hemodynamic events between the groups, occurrence of nausea and vomiting, and incidence of delirium assessed by the CAM-ICU tool in the PACU between the groups). The analysis was performed on a per-protocol approach, but a sensitivity analysis was additionally performed based on the intention-to-treat for the opioid consumption including the primary outcome. The data are expressed as the mean ± standard deviation, mean ± standard deviation [95% confidence interval (CI)], or the number of patients (%). Serially measured variables, including NRS for pain in the PACU and modified Aldrete scores, were analyzed by performing repeated measures analysis of variance. NK cell activity and IL-6 levels were log-transformed for normality of distribution and analyzed using paired t-test. Statistical significance was set at *P* < 0.05.

## Results

Seventy-five patients (38 in the SPI group and 37 in the conventional group) completed this study (Fig. 1). There was no significant difference in patient characteristics, baseline clinical data, anesthesia time, and the types of operation between the two groups (Table 1).

**Table 1:** Characteristics and clinical data of patients

	Conventional group (*n* = 37)	SPI group (*n* = 38)
Age (years)	74.6 ± 6.5	74.5 ± 5.2
Weight (kg)	56.9 ± 12.1	59 ± 10.2
Height (cm)	157.4 ± 10.3	159.2 ± 8.3
Sex (M/F)	17/20	23/15
ASA physical status (I/II/III)	0/30/7	0/32/6
Diabetes mellitus (no/yes)	28/8	29/9
Hypertension (no/yes)	20/16	15/23
Medication (no/yes)		
Beta blocker	35/2	30/8
Calcium channel blocker	27/10	24/14
ARB or ACEi	28/9	27/11
Preoperative chemotherapy (no/yes)	32/5	33/5
Anesthesia time (min)	171.0 ± 46.6	169.1 ± 52.9
Operation type		
Hemicolectomy	9	8
Anterior resection	14	16
Low anterior resection	11	10
Others^a^	3	4

Values are mean ± SD or number

*SPI* surgical pleth index, *ASA physical status* American Society of Anesthesiologists physical status, *ARB* Angiotensin II receptor blocker, *ACEi* Angiotensin converting enzyme inhibitor

^a^Others in the operation type included segmental resection, and subtotal/total colectomy

The intraoperative remifentanil consumption (average infusion rate) was significantly higher in the SPI group than in the conventional group (0.13 ± 0.05 μg/kg/min [95% CI, 0.11–0.14] vs. 0.06 ± 0.04 μg/kg/min [95% CI, 0.05– 0.08], *P* < 0.001) (Table 2). Regarding the measurement of hemodynamic parameters, the total number of BP and HR measurements during anesthesia was 1285 in the SPI group and 1266 in the conventional group. The incidence of hypertension events in the SPI group was 14 (1.1%), while that in the conventional group was 35 (2.8%) (*P* = 0.002). The incidence of tachycardia events in the SPI group was 15 (1.2%), while that in the conventional group was 50 (3.9%) (*P* < 0.001). The incidence of hypotension and bradycardia events in the SPI group was similar to that in the conventional group (Table 2). The awakening time was similar between the groups (Table 2).

**Table 2:** Remifentanil consumption and hemodynamic events during surgery, and awakening time

	Conventional group (*n* = 37)	SPI group (*n* = 38)	*P*-value
Remifentanil consumption (μg/kg/min)	0.06 ± 0.04	0.13 ± 0.05	< 0.001
Hemodynamic episodes^a^			
Incidence of tachycardia (%)	3.9	1.2	< 0.001
Incidence of bradycardia (%)	0.95	1.1	0.72
Incidence of hypertension (%)	2.8	1.1	0.002
Incidence of hypotension (%)	1.7	1.6	0.83
Awakening time (sec)	513.3 ± 185.6	442.6 ± 217.6	0.13

Values are mean ± SD or percentage of episodes or number of patients (%). SPI: surgical pleth index

^a^The total number of measurements of heart rate (HR) and blood pressure during anesthesia was 1266 in convetional group and 1285 in SPI group. Tachycardia was defined as HR > 90 beats/min. Hypertension was defined as mean arterial pressure (MAP) > 100 mmHg, if the baseline MAP was below 83 mmHg, or as an increase of over 20% of the baseline MAP, if it was higher than 83 mmHg. Bradycardia was defined as HR < 45 beats/min. Hypotension was defined as MAP < 60 mmHg

**Fig. 2:**
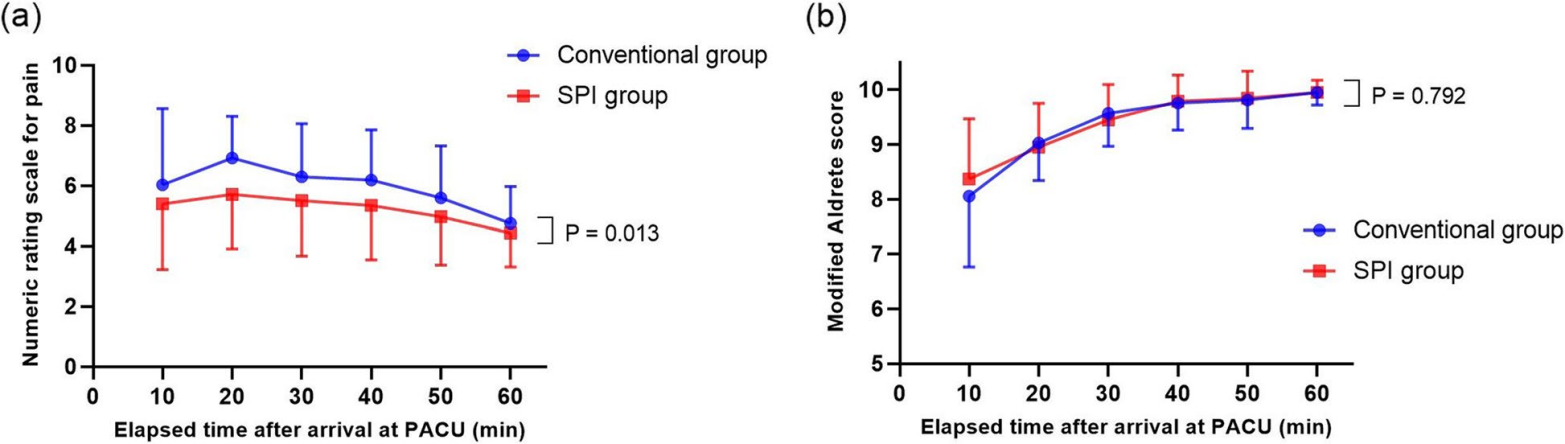
Changes over time in (**a**) numerical rating scale (NRS) for pain and (**b**) modified Aldrete score at post-anesthesia care unit (PACU) in the conventional group or the SPI group. The graph shows a mean and standard deviation of (**a**) NRS for pain and (**b**) modified Aldrete score for each time point

In the PACU, the change over time in modified Aldrete score at PACU was comparable between the groups (*P* = 0.792; Fig. 2b). However, the change over time in NRS for pain at PACU was higher in the conventional group than in the SPI group (*P* = 0.013; Fig. 2a). Fentanyl consumption in the PACU was higher in the conventional group than in the SPI group, although the difference was not statistically significant (70.3 ± 35.8 μg [95% CI, 58.3–82.2] vs. 55.9 ± 29.3 μg [95% CI, 46.1–65.7]; *P* = 0.06). The incidence of delirium in the PACU was significantly lower in the SPI group than in the conventional group (5.2% vs. 24.3%, *P* = 0.02). Nausea or vomiting in PACU, NRS for pain on POD 1, cumulative pethidine consumption on POD 1 (70.9 ± 13.8 mg [95% CI, 66.3–75.6] vs. 62.5 ± 22.3 mg [95% CI, 55.2–69.8]; *P* = 0.074) were comparable between the two groups, as was the postoperative length of hospital stay (Table 3).

**Table 3:** Incidence of nausea or vomiting, and delirium, fentanyl consumption at post-anesthesia care unit (PACU), and other postoperative data

	Conventional group (*n* = 37)	SPI group (*n* = 38)	*P*-value
Nausea or vomiting in PACU (n)	3 (8.1%)	1 (2.6%)	0.29
PACU fentanyl consumption (μg)	70.3 ± 35.8	55.9 ± 29.3	0.06
Delirium assessed by CAM-ICU (n)	9 (24.3%)	2 (5.2%)	0.02
NRS for pain in POD 1 (0–10)	3.0 ± 0.75	2.9 ± 0.46	0.989
Cumulative pethidine consumption in POD 1 (mg)	70.9 ± 13.8	62.5 ± 22.3	0.074
Postoperative length of hospital stay (day)	10.4 ± 9.1	8.0 ± 2.1	0.120

Values are number of patients (proportion) or mean ± SD

*SPI* surgical pleth index, *PACU* Post-anesthesia care unit, *CAM-ICU* Confusion assessment method for the intensive care unit, *NRS* numerical rating scale, *POD* Postoperative day

No significant difference was noted in the change in NK cell activity and serum interleukin-6 levels during the perioperative period between the two groups (Table 4).

**Table 4:** Perioperative values of interleukin-6 (IL-6) and natural killer (NK) cell activity

Variable/ measurement time	Conventional group (*n* = 37)		SPI group (*n* = 38)		*P*-value
	Log-transformed	Raw	Log- transformed	Raw	
IL-6 (pg/mL)					
Before surgery	1.35 ± 1.13	7.25	1.25 ± 1.07	8.20	0.606
POD 1	3.85 ± 1.06	77.33	3.80 ± 1.01	78.24	
NK cell activity (pg/mL)					
Before surgery	4.95 ± 1.02	262.30	5.06 ± 1.22	348.20	0.326
POD 1	3.29 ± 0.96	41.38	3.76 ± 1.06	110.62	

Values are mean ± SD

*SPI* surgical pleth index, *POD* Postoperative day

The intention-to-treat analysis included 43 patients in the conventional group and 42 patients in the SPI group (withdrawal after surgery due to patient refusal was excluded). The results were similar with the primary per-protocol analysis as follows. The SPI group consumed significantly more intraoperative remifentanil (average infusion rate) than the conventional group (0.13 ± 0.05 μg/kg/min [95% CI, 0.12–0.15] vs. 0.07 ± 0.05 μg/kg/min [95% CI, 0.06–0.09], *P* < 0.001). Fentanyl consumption in the PACU was higher in the conventional group than in the SPI group (71.5 ± 33.9 μg [95% CI, 61.0–81.9] vs. 58.9 ± 31.1 μg [95% CI, 49.2–68.6]; *P* = 0.051). Cumulative pethidine consumption on POD 1 was comparable between the two groups (70.9 ± 15.3 mg [95% CI, 66.2–75.7] vs. 63.7 ± 22.2 mg [95% CI, 56.8–70.6]; *P* = 0.138).

## Discussion

In this study, we found that in elderly patients, SPI-guided remifentanil administration during laparoscopic colorectal cancer surgery under general anesthesia was significantly higher than that in the conventional analgesia practices, resulting in lower intraoperative hypertension and tachycardia events without compromising postoperative pain and recovery in the PACU.

These results suggest several important points regarding SPI-guided analgesia in elderly patients. First and foremost, contrary to the hypotheses for our study, the results differ from those of previous studies [5, 9, 13–15]. Unlike previous studies that found less intraoperative opioid consumption in the SPI-guided group consisting of healthy adults over the age of 20 years, the SPI-guided group in this study received significantly more remifentanil than the control group.

The cardiovascular response to pain appears blunted in the elderly during general anesthesia for reasons such as decreased myocardial contractility, myocardial and vascular elasticity, baroreceptor reflex activity, and β-adrenergic response [16, 17]. Moreover, blood pressure can be suppressed during volatile anesthesia by both direct and indirect effects on the autonomic nervous system affecting the heart [2, 18, 19], which can lead to aggressive hypotension in elderly patients. As a result of these factors, the degree of pain in some of the conventional group patients might be masked, resulting in insufficient analgesic consumption during general anesthesia. However, SPI might enable adequate analgesia in elderly patients as it was appropriately reflected in the degree of pain (despite increased vascular stiffness and decreased vascular elasticity in those patients).

PPGA and HBI influence the SPI value, and the PPGA is affected by vascular wall distensibility and intravascular pulse pressure [20]. Age-related changes in vascular structure and function (i.e., less elastin and advanced glycation end-products) cause an exacerbation of small and large arterial stiffness, a characteristic change associated with older age [21], so that the delivery of pressure waves is accelerated and the intensity is increased. Changes (decreases) in PPGA from sympathetic stimulation are likely to increase with age [22–24]. This is the opposite response seen in pediatric patients with a lower vascular tone and higher vascular distensibility than in adults; unlike adults, pediatric patients are less likely to show prominent decreases in PPGA from sympathetic stimulation, resulting in an underestimation of the SPI value [20]. Therefore, for proper analgesia during surgery in pediatric patients, analgesics should be administered when the SPI is < 40 rather than the range of 20–50 (the reference value) that is recommended for adults [20, 25, 26]. Conversely, SPI may be overestimated in older adults because the PPGA from sympathetic stimulation decreases with age. The increased SPI value caused by the decrease in the PPGA may be offset by the less change in the HBI, because the HBI response is not appropriately reflected in older patients due to blunted cardiovascular response [16, 17].

As a result, these characteristics may keep the SPI target range of 20–50 to properly reflect intraoperative nociception in patients of this study, resulting in more remifentanil consumption in the SPI group than in the control group. Similarly, due to the blunted autonomic responses, conventional analgesia responding to hemodynamic response can be under-dosed in the elderly patients. In contrast, SPI guided analgesia may provide a more appropriate level of analgesia with sufficient remifentanil dose.

Second, In the SPI-guided analgesia group, there was less postoperative pain and analgesic consumption in the PACU as a result of the appropriate analgesia during surgery and the incidence of delirium in the PACU was significantly lower. These findings suggest that reducing pain during and after surgery, via intraoperative SPI-guided analgesia may be a factor that can reduce postoperative delirium [27, 28], thereby affecting the prognosis in elderly patients immediately after surgery. The novel finding for postoperative delirium may require further validation in future studies that include long-term follow-up investigations.

Third, our study differs from previous studies in that it excluded patients with cardiovascular disease or those taking medications that interfered with the cardiovascular response and prevented SPI values based on PPGA and HBI from deteriorating. However, most elderly patients have comorbidities, such as DM, hypertension, and coronary artery disease, without arrhythmia [11, 29, 30]. Managing the nociceptor activation-anti-nociception balance in such patients is more important. Therefore, we included older patients with these comorbidities and identified their medications. Because the elderly patients had underlying diseases and were taking drugs such as calcium channel blockers, beta-blockers, angiotensin II receptor blockers, and angiotensin-converting enzyme inhibitors, the BP and HR during surgery may not reflect the response to the patient’s nociceptor stimulation under the influence of these drugs. If the analgesic dose is adjusted while the BP and HR are monitored using conventional methods, these patients may not have an adequate analgesic effect. Indeed, our findings demonstrated the usefulness of SPI monitoring in these patients.

Surgical stress is characterized by an inflammatory response with immune suppression, and surgical trauma initiates a cascade of reactions involving the immune response and nociception [31]. In this regard, we anticipated that SPI-guided analgesia could modulate deteriorated immune function after surgery by attenuating stress response. However, the perioperative changes in NK cell activity and IL-6 showed no significant difference between the two groups. Although there was a significant difference in intraoperative remifentanil consumption between the two groups, all of them received a relatively low dose of remifentanil. The postoperative immune blood test was performed only on the first day of the operation rather than at other time points, including the end of the surgery or at PACU. There was no difference in NRS for pain on POD 1 between the two groups. Consequently, the sample timing and protocol for postoperative pain management in this study are thought to have an impact on the results. Furthermore, not only does the level of stress and nociception during surgery affect perioperative NK cell activity (cytotoxicity) or cellular immunity to varying degrees, but inhalational anesthetics (e.g., sevoflurane) and opioids can have a variable effect [32–35]. However, Cronin et al. reported that low-dose remifentanil infusion did not impair NK cell activity in healthy volunteers [36], suggesting that intraoperative remifentanil consumption in this study might not be a factor for immunomodulation in the perioperative period. Various factors can influence the perioperative immune and inflammatory response, complicating the prediction of relevant outcomes. Therefore, further studies are needed to investigate the effect of SPI-guided analgesia on the perioperative inflammatory and immune response.

This study has several limitations. First, because of the study design, staff in the operation room could not be blinded to the group assignment. However, the investigators who conducted follow-ups outside the operating room, including laboratory investigations and postoperative data assessment, were unaware of the patient’s group assignment. Second, the vasoactive agents (ephedrine, phenylephrine, and nicardipine) used in this study may affect SPI values by alternating the PPGA and HBI, which may interfere with the interpretation of proper SPI values [37]. Third, hemodynamic management based on (static or dynamic) hemodynamic monitoring, such as central venous pressure, cardiac output, or stroke volume (or its variation), may be useful for fluid and vasopressor management. However, accurate values of these variables usually require invasive monitoring. In colorectal surgery, where invasive monitoring is not routinely required, changes in blood pressure and heart rate may be achieved through conventional methods. Fourth, the population size had potential implications because the study size was estimated using different assumptions than the actual results.

## Conclusions

In elderly patients undergoing laparoscopic colorectal cancer surgery under general anesthesia, SPI-guided remifentanil consumption was significantly higher than that of conventional analgesia with lower intraoperative hypertension and tachycardia events, and a lower incidence of delirium in the PACU. Nevertheless, perioperative changes in NK cell activity and IL-6, did not show a significant difference between the two groups. Therefore, we suggest that in the elderly undergoing laparoscopic colorectal cancer surgery, SPI-guided analgesia may provide appropriate analgesia with reduced intraoperative hypertension and tachycardia and improve postoperative outcomes, including postoperative pain and delirium in the PACU.

## Data Availability

All data generated or analyzed during this study were included in this published article.

## Abbreviations

SPI: Surgical pleth index
PACU: Post-anesthesia care unit
NK: Natural killer
IL: Interleukin
HBI: Heart beat interval
PPGA: Photoplethysmographic waveform amplitude
HR: Heart rate
BP: Blood pressure
MAP: Mean arterial pressure
ASA: American Society of Anesthesiologists
TCI: Target controlled infusion
Ce: Effect-site concentration
NRS: Numerical rating scale
CAM-ICU: Confusion assessment method for the intensive care unit
POD: Postoperative day
DM: Diabetes mellitus
